# Supplementary material for: Two Auxin Response Elements Fine-Tune PINOID Expression During Gynoecium Development in Arabidopsis thaliana
Source: Biomolecules. 2019 Sep 25;9(10):526. doi: 10.3390/biom9100526 (PMC6843594; doi:10.3390/biom9100526)
Supplement: Supplementary file 1 [file biomolecules-09-00526-s001.pdf]

biomolecules

MDPI

Article

Two Auxin-Response Elements Fine-Tune *PINOID* Expression During Gynoecium Development in *Arabidopsis thaliana*

André Kuhn, Bethany Runciman, William Tasker-Brown and Lars Østergaard *

Department of Crop Genetics, John Innes Centre, Norwich Research Park, Norwich NR4 7UH, United Kingdom; andre.kuhn@jic.ac.uk (A.K.); b.runciman@aol.co.uk (B.R.); billy.tasker-brown@jic.ac.uk (W.T.-B.)
* Correspondence: lars.ostergaard@jic.ac.uk

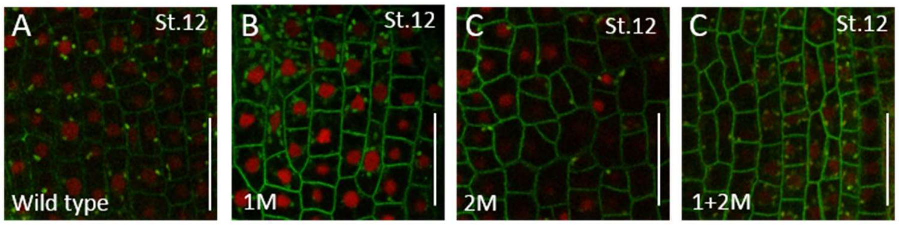

Figure S1. PID-GFP localises to the plasma membrane in an apolar fashion in all rescue promoter variants. (A-D) Representative confocal images of the style region of stage 12 for the respective promoter variant reporter lines. Scale bars = 20 μm.

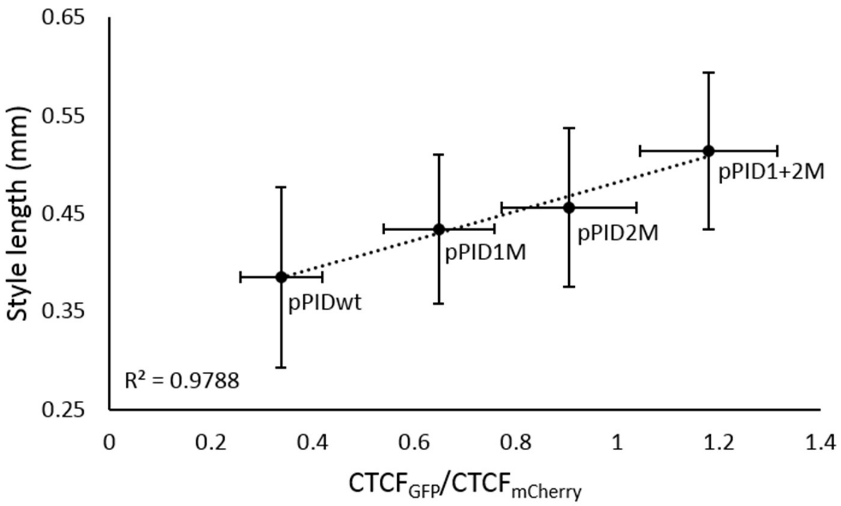

Figure S2. Correlation between PID promoter activity at stage 12 and style length in different AuxRE mutant lines. Shown are means ± standard deviation.

Table 1. List of Primers used for cloning.

ID	Orientation	Description	Sequence 5′-3′
**AK173**	Sense	Promoter	TGAAGACTTGGAGCGTGTAGACCAAATCCAGGGT
**AK174**	Antisense	Promoter	TGAAGACTTCATTCGCCGGGAAAATCGAAGTTA
**AK175**	Antisense	Promoter 1M	TGAAGACTTTGAGACGTTATCACGTGCCTGAAA
**AK176**	Sense	Promoter 2M	TGAAGACTTCTCACACGTGTCATATATCTTACG
**AK177**	Sense	Promoter 1M	TGAAGACTTCTCACACGTATCATATATCTTACG
**AK178**	Antisense	Promoter 2M	TGAAGACTTTGAGACGTTGTCACGTGCCTGAAA
**AK179**	Sense	Gene part1	TGAAGACTTAATGTTACGAGAATCAGACGGTGAGA
**AK180**	Antisense	Gene part1	TGAAGACTTGCCTCCTGATCTCTGCGTAAGCGA
**AK181**	Sense	Gene part2	TGAAGACTTAGGCGAAAAAAACAAGGCCTAACC
**AK182**	Antisense	Gene part2	TGAAGACTTATGACGAGGAAGATTCAACGGCTG
**AK183**	Sense	Gene part3	TGAAGACTTTCATCGCCGGAGAATCAACAACTC
**AK184**	Antisense	Gene part3	TGAAGACTTCGAAGATCCAAAGTAATCGAACGCCG
**SS-IND**	Sense	IND	GGAAGACTTAATGGAGCCTCAGCCTCACCATCTCC
**SS-IND**	Antisense	IND	GGAAGACTTCGAACCGGGTTGGGAGTTGTGGTAATAAC
**SS-pIND**	Sense	pIND	GGAAGACGGGGAGGAAAGTGTGTAATCCAAATCGTTTGCG
**SS-pIND**	Antisense	pIND	GGAAGACGGCATTTTTCATCTTTTCTTATTTCTC

Table 2. List of plasmids used for cloning. The Plasmids are available from TSL Synbio (http://synbio.tsl.ac.uk/).

ID	Level	Description	Comments	Selection
**pICH41295**	L0	Acceptor for Pro+5U modules	AddGene #47997	Spectinomycin
**pICSL01005**	L0	Acceptor for CDS no stop (ns) modules	AddGene #47996	Spectinomycin
**pICSL50005**	L0	Yellow Fluorescent Protein (YFP) for C-terminal fusion	-	Spectinomycin
**pICSL50008**	L0	Green Fluorescent Protein (GFP) for C-terminal fusion	AddGene #50314	Spectinomycin
**pICSL80013**	L0	GFP with Simian Virus 40 nuclear localisation signal	-	Spectinomycin
**pICH50581**	L0	ACTIN 2 Constitutive promoter – plants	AddGene #50256	Spectinomycin
**pAGM5331**	L0	Simian Virus 40 nuclear localisation signal	AddGene #50294	Spectinomycin
**pICSL80007**	L0	mCherry Fluorescent protein	AddGene #50321	Spectinomycin
**pICH41421**	L0	Nos (nopaline synthase) terminator	AddGene #50339	Spectinomycin
**pICSL11017**	L1	Position1 BASTA resistance cassette	-	Ampicilin
**pICH47742**	L1	Position 2	AddGene #48001	Ampicilin
**pICH47751**	L1	Position 3	AddGene #48002	Ampicilin
**pICH41766**	L1	End-linker 3 for level 2 construction	AddGene #48018	Ampicilin
**pAGM4723**	L2	Binary vector	-	Kanamycin
